# Editorial: Towards an integrated approach to measurement, analysis and modeling of cortical networks

**DOI:** 10.3389/fncir.2015.00061

**Published:** 2015-10-20

**Authors:** A. Ravishankar Rao, Guillermo A. Cecchi, Ehud Kaplan

**Affiliations:** ^1^Gildart Haase School of Computer Sciences and Engineering, Fairleigh Dickinson UniversityTeaneck, NJ, USA; ^2^IBM Thomas J. Watson Research CenterYorktown Heights, NY, USA; ^3^Icahn School of Medicine at Mount SinaiNew York, NY, USA

**Keywords:** cortical networks, neural dynamics, graph measures, emergent properties

## 1. Introduction

Recent technological advances have led to an unprecedented increase in the volume and detail of neuroscientific data, creating significant challenges for their processing and interpretation. We approach this challenge through a network-centric perspective, as we believe that brain function is fundamentally determined by patterns of connectivity between neurons, and the resulting dynamics. This is in contrast to traditional computational neuroscience techniques that focus on models of individual neurons and compartments. Progress, in consequence, is essential on (at least) three major fronts: *measurement* of neural activity, *analysis* of network structures deduced from this activity, and *modeling* of network function, leading to theoretical insights.

The *measurement* front spans the range from multi-electrode recordings to whole-brain measurements using imaging. Several basic scientific questions arise: What do we need to measure in brain networks? Are there theoretical constraints that would dictate this? How do we design our experiments to generate the most meaningful data? How do we record from awake/behaving animals, or even from multiple animals interacting socially?

The *analysis* front consists of creating network models from the measurements. Some promising techniques explore the estimation of networks using causality. However, several open questions remain: How do we define the fundamental units within the network? Are these units fixed or do they evolve dynamically? How do we infer connectivity between network elements? How do we identify functional clustering, based on the individual neuronal features? How do we quantify and interpret the activity of multiple neurons via multi-unit recordings, especially when there is no stimulus-response paradigm?

The *modeling* front can proceed in several directions. From the extracted network we can identify topological regularities, such as motifs and cycles. An interesting research direction is to analyze the relationship between the structure of the network, as represented by its motifs, and its function. A growing body of work is examining the relationship between network structure and phenomena such as stability and synchrony. For instance, neurons in the hippocampus could be modeled as a network wherein hubs consisting of hub neurons promote synchrony, while cycles in this network may cause instability. The theme of synchrony as an important network phenomenon emerges in several articles in this research topic (Canavier et al., 2013; Latorre et al., 2013; Tibau et al., 2013; Vardi et al., 2013; Cavallari et al., 2014; Chary and Kaplan, 2014; Konstantoudaki et al., 2014; Ratnadurai-Giridharan et al., 2014).

We emphasize that the three fronts consisting of measurement, analysis and modeling are interdependent, but must evolve synergistically. The model and theoretical understanding need to be grounded in constraints produced by the measurement process. Insights derived from modeling can be used to drive novel experiments and measurement techniques. An emerging trend deploys active probing and network manipulation through viral vectors and optogenetic methods.

We expect that by aligning existing and future research along these fronts, we will be able to answer questions at the system level. We can view this development as a generalization of the Hubel-Wiesel approach which characterizes feed-forward sensory coding to approaches that characterize dynamic network-level interactions with the input signals. We can derive value from our understanding of network function by applying it to brain-related disorders, such as schizophrenia, drug addiction, or autism. For instance, differences between default mode networks of ASD (autism spectrum disorder) subjects and normals have been reported, among other psychiatric and neurodegenerative conditions. Cortical network properties ultimately determine how different network oscillation states are established and maintained and defining these principles could explain why there is impaired synchronization between different brain areas in schizophrenics and Parkinson's patients. Overall, network-based measures capture better the dynamics of brain processes, and provide features with greater discriminative power than point-based measures.

The articles in this research topic cover these different aspects of cortical networks. To guide the reader, we provide below a brief summary of each article, and relate it to the overall theme of the research topic.

## 2. Mechanistic models of neuronal dynamics

Rothganger et al. (2014) present a model design platform, N2A, which has the potential to speed up the process of designing and validating biologically realistic models. By utilizing a hierarchical representation of neural information, N2A allows models from different users to be combined. N2A natively implements standard computations in sensitivity analysis and uncertainty quantification, which allows users to validate models easily. They demonstrate the versatility of N2A through several examples.

Ratnadurai-Giridharan et al. (2014) develop a biophysically relevant network model of the CA1 subfield, and investigate the relationship between network properties and the susceptibility of CA1 to exhibit interictal spikes (IIS). They investigate the conditions under which synchronization of paroxysmal depolarization shift (PDS) events evoked in CA1 pyramidal (Py) cells can trigger an IIS. Like other papers in this research topic, they explore the conditions necessary for and consequences of synchrony, and find that spontaneous IISs closely depend on the degree of the network's intrinsic excitability.

Bhattacharya et al. (2014) present a study of a thalamo-cortico-thalamic (TCT) implementation on SpiNNaker (Spiking Neural Network architecture), a hardware platform inspired by the processing parallelism, and energy efficiency of biological neural networks. Their system presents similar dynamic and spectral features to EEG in the sleep-wake transition, and could lead to much larger TCT models.

## 3. Descriptive and model-based measurements of experimental data

Dey et al. (2014) use Resting State fMRI functional connectivity and a combination of topological and neuroanatomical features to implement predictive modeling on a dataset of Attention Deficit Hyperactive Disorder (ADHD) and control subjects, and obtain a high predictive accuracy, over 70 for 50% chance. The use of graph-theoretic and anatomical features emphasizes the notion that different brain functions (and dysfunctions) are an emergent property of the interaction between specific brain areas.

Alonso et al. (2014) test a specific hypothesis derived from theorizing the brain as a system determined by emergent properties, namely dynamical criticality. Studying ECoG recordings of anesthesia induction in humans, they show that depth of anesthesia is concomitant with increased dynamical stability, as estimated by the eigenvalues of fitted moving-window auto-regressive models. They further demonstrate that this stabilization effect cannot be explained by the spectral changes associated with anesthesia, which are typically used to characterize the transition to unconsciousness.

Almeida-Filho et al. (2014) study multi-electrode recordings in the hippocampus and early visual and sensory cortices of rats during and after novel object exploration, as well as during the sleep cycle. They identified cell assemblies as a linear combination of the units’ activity, and determined phase relationships between these assemblies. They computed a graph whose nodes correspond to assemblies, and edges correspond to phases. They use graph-theoretic features to perform high accuracy predictive modeling with a simple classifier (Naive Bayes).

Vardi et al. (2013) propose a mechanism that allows time-lags among populations of spiking neurons to drop from several tens of milliseconds to nearly zero-lag synchrony. The mechanism allows sudden leaps out of synchrony, hence creating short epochs of synchrony. They obtained results by enforcing conditioned stimulations on neurons embedded within a large cortical network *in vitro*. Their simulations support the proposed underlying biological mechanisms: the increase of neuronal response latency to ongoing stimulations and temporal or spatial summation required to generate evoked spikes.

Tibau et al. (2013) monitored the development of neuronal cultures, and recorded their activity using calcium fluorescence imaging. They demonstrate that the power spectrum can be used as a signature of the state of the network, for instance, when inhibition is active or silent, as well as a measure of the network's connectivity strength. The power spectrum identifies prominent developmental changes in the network, and reveals the existence of communities of strongly connected, highly active neurons that display synchronous oscillations. Using this approach, one could distinguish healthy from diseased networks, or track the effects of therapeutic interventions.

Riera et al. (2014) describe an “electro-physiological microscope” with high spatial and temporal resolution. It consists of a 3-dimensional array of micro-electrodes, and a novel way of analyzing the current-source density data collected by the array. Their method can localize single whisker barrels from event-related responses to a single whisker deflection, but can also provide information about the spatiotemporal dynamics of neuronal aggregates in several barrels, with the resolution of single neurons. Their method constitutes a significant advance over previous approaches, and could thus change the way the activity of cortical neurons is analyzed in the future.

## 4. Network functionality

### 4.1. The importance of being synchronized

For the past several decades, theoreticians and experimentalists alike have focused on neuronal synchrony and on the important roles that it might play in brain function, from “The Binding Problem” in perception (Gray, 1999) to Consciousness (Crick and Koch, 1990; Melloni et al., 2007). For a fuller discussion and additional references, see Singer (2007). Several of the articles in this research topic illuminate the issue of synchrony from both physiological and computational perspectives.

Canavier et al. (2013) address the problem of how neurons can synchronize their responses with minimal time lag. They developed a graphical method for determining the effect of the phase response curve (PRC) shape on synchronization and illustrate it using type 1 PRCs, consisting of advances (delays) in response to excitation (inhibition). They showed that the skewness of the PRC affects synchrony. Their analysis of pairwise synchronization tendencies form a useful framework to understand the synchronization behavior of neurons within larger networks.

Konstantoudaki et al. (2014) explore the role of interneurons in the maintenance of a dynamic balance between excitation and inhibition, since changes in this balance have been identified in several neuropsychiatric diseases, such as schizophrenia. They constructed a pre-frontal-cortex (PFC) microcircuit, consisting of pyramidal neuron models and the three interneuron types described in the literature. Their simulations showed that generic somatic inhibition acts as a pacemaker of persistent activity, and that fast-spiking specific inhibition modulates the amplitude and synchrony of the pacemaker's output.

Nie et al. (2014) make use of Information Geometry (IG), which is based on the expansion of the joint probability distribution of an N-neuron system. They used two measures, the single-IG measure and the pairwise IG-measure to examine the activity of simulated interconnected neurons that exhibit oscillations. They considered two oscillatory mechanisms, externally driven oscillations and internally induced oscillations. For both mechanisms, they showed a linear relationship between the single-IG measure and the external input magnitude and a linear relationship between the pairwise-IG measure and the the sum of connection strengths between two neurons.

Cavallari et al. (2014) investigate the effect of employing current- or conductance-based synapses in models of neural networks, both of which have been widely used. They create comparable networks that use the two types of synapses, and compare their dynamics. They report that these two types of networks, which had comparable first-order statistics, showed profound differences in their second-order statistics of neural interactions, and in the modulation of these properties by external inputs. Thus, the second order statistics of the network dynamics depend strongly on the choice of synaptic model, a fact that modelers of neural networks will find very useful.

Thivierge et al. (2014) investigate synaptic motifs created by a relay network, where two populations of neurons communicating via a third relay population achieve synchronization. By employing models of neuronal dynamics, they demonstrate that the use of relay networks leads to the creation of a global attractor of activity that prevents neurons from being responsive to input stimuli. They overcome this limitation by introducing a selective gain inhibition mechanism which allows neurons to respond effectively to external stimuli. They present results to show that patterns of neural synchronization follow stimulus presentation, and that synchronization disappears after the stimulus is removed.

Chary and Kaplan (2014) investigate the role of synchrony in the functioning of reward circuits in the brain. Their computational study demonstrates that synchrony can have two opposing effects in networks that are sensitive to the correlation between stimulus and reward: weakly correlated inputs amplify short-term recall, but suppress long-term recall. Their main finding is that even weak stimulus-reward correlations can facilitate the short-term repetition of a pattern of neural activity, while blocking the long-term embedding of that pattern.

Latorre et al. (2013) implement a network model of the Inferior Olive (IO) to study its synchronization behavior, using electrically coupled conductance-based neurons. In the presence of stimuli, different rhythms are encoded in the spiking activity of the IO neurons that nevertheless remains constrained to a commensurate value of the subthreshold frequency. Moreover, the stimuli induced spatio-temporal patterns that reverberate for long periods. These results have implications beyond IO studies, and is related to tremor, migraine, and epilepsy where these modeling techniques could have a potentially significant impact.

### 4.2. Computation

Kaplan and Lansner (2014) address the issue of odor perception, and investigate the processing of odors through multiple processing stages within a hierarchical system. They use a large-scale network model which spans olfactory receptor neurons (ORNs), three types of cells in the olfactory bulb, and three types of cortical cells in the piriform cortex. A competitive Hebbian–Bayesian learning algorithm is used to adjusting synaptic weights. Their model is able to perform robust concentration-invariant odor recognition.

Eguchi et al. (2014) use a detailed computational model of the early visual system in an attempt to bring our understanding of cortical color processing to a level thought to exist for orientation processing. They use information-theoretic measures, and train their model using natural images, in trying to understand how cells of similar color preference come to cluster together in the cortex. Like several other papers in this research topic they also explore the function of synchrony, and the role it might play in deciding what color is used in the visual stimulus.

### Conflict of interest statement

The authors declare that the research was conducted in the absence of any commercial or financial relationships that could be construed as a potential conflict of interest.
